# Metabolic Modifications, Inflammation, and Cancer Immunotherapy

**DOI:** 10.3389/fonc.2021.703681

**Published:** 2021-09-24

**Authors:** Sihao Zheng, Qibin Song, Pingfeng Zhang

**Affiliations:** Cancer Center, Renmin Hospital of Wuhan University, Wuhan, China

**Keywords:** metabolic modifications, glycosylation, palmitoylation, inflammation, cancer immunotherapy, PD-1/PD-L1

## Abstract

Cancer immunotherapy has accomplished significant progresses on treatment of various cancers in the past decade; however, recent studies revealed more and more heterogeneity in tumor microenvironment which cause unneglectable therapy resistance. A central phenomenon in tumor malignancy is metabolic dysfunctionality; it reprograms metabolic homeostasis in tumor and stromal cells thus affecting metabolic modifications on specific proteins. These posttranslational modifications include glycosylation and palmitoylation, which usually alter the protein localization, stability, and function. Many of these proteins participate in acute or chronic inflammation and play critical roles in tumorigenesis and progression. Therefore, targeting these metabolic modifications in immune checkpoints and inflammation provides an attractive therapeutic strategy for certain cancers. In this review, we summarize the recent progresses on metabolic modifications in this field, focus on the mechanisms on how glycosylation and palmitoylation regulate innate immune and inflammation, and we further discuss designing new immunotherapy targeting metabolic modifications. We aim to improve immunotherapy or targeted-therapy response and achieve more accurate individual therapy.

## Introduction

In addition to conventional cancer therapies such as surgery, radiotherapy, chemotherapy, and targeted therapy, immunotherapy is playing a more and more important role in cancer treatment. Several types of modern cancer immunotherapies have been developed in the past decades, including immune checkpoint therapy (ICT), adoptive cell therapy, and cancer vaccines, especially since the first immune checkpoint inhibitor ipilimumab was developed and approved by the Food and Drug Administration (FDA) in 2011 (1, 2). The cancer cells reprogram their metabolic pathways to adapt to the tumor microenvironment and suppress the immune system which is supposed to recognize the cancer cells (through cancer antigens) and attack them. Cancer immunotherapy harnesses the patients’ own immune system to target cancer cells, thus it has high specificity and efficacy and has already achieved significant outcomes in clinical practice on certain cancer types (3, 4). Immune checkpoints are molecules on certain immune cells that need to be activated (or inactivated) to license an immune response. Since 2011, several immune checkpoint inhibitors have been approved by the US FDA for second- and even first-line treatment against various cancer types, such as lung cancer, renal cell carcinoma, bladder cancer, gastric cancer, and many others; these include the CTLA-4–blocking antibody ipilimumab (5), three antibodies against Programmed Cell Death Receptor-1 (PD-1, CD279) [pembrolizumab (6), nivolumab (7), and cemiplimab (8)], and three antibodies targeting Programmed Cell Death Ligand-1 (PD-L1, CD274) [atezolizumab (9), avelumab (10), and durvalumab (11)]. An increasing number of clinical trials have proven that using antibodies targeting immune checkpoints has longer follow-up than other agents and leads to improved survival with durable clinical response that can last more than a decade for patients with advanced carcinoma (1, 12–15).

Despite significant advances in clinical practice, ICT still faces outstanding challenges that need to be addressed urgently. First of all, ICT is not effective against all cancer types, furthermore, even within a “responsive” cancer type, not every patient is responsive, thus it is difficult to determine which cohort of patients should be suitable for ICT. Taking pharmaceutical treatment of PD-1 as an example, clinical data showed that only about 20% PD-L1–positive NSCLC (which accounts for 85% of all lung cancers) patients respond objectively to ICT (16, 17). The reason of the phenomenon called *de novo* resistance mainly encompasses immune evasion by other checkpoint pathway and incapable of T-cell infiltration to the tumor microenvironment, which is linked to innate immunity especially inflammation. Surprisingly, several clinical trials reveal that the survival benefits for NSCLC patients from anti-PD1/PD-L1 immunotherapy is independent of PD-L1 expression (18–23), thus the true predictive value of PD-L1 is limited (24). This may attribute to the failure of accurate measurement and scoring of PD-L1 protein expression with antibodies. Secondly, a substantial proportion of patients who initially respond to ICT ultimately relapse with lethal, drug-resistant disease months or years later, which is referred to as acquired resistance. Recent studies suggested that main determinants of acquired resistance are consist of function of interferon signaling pathways (25, 26), expression of antigen-presenting molecules (27), and immune-evasive oncogenic signaling pathways (28). In addition, ICT also elicits inflammatory side effects, which are often termed immune-related adverse events and primarily harm organs including lung, gastrointestinal tract, endocrine glands, skin, and liver (29).

Emerging data indicated that traditional single drug treatment is not sufficient for improving the progression-free survival of patients with tumor, and the efficacy of therapy in combination with anti-CTLA4, anti-PD1, or anti-PDL1 antibodies, as well as with other immunotherapies, chemotherapies, radiation therapies, and targeted therapies is better than that of treatment with single drug. Indeed, combinations of chemotherapy and/or targeted therapy with ICT have been approved as first-line treatments for several conditions, including lung cancer and advanced renal cell carcinoma (30, 31). However, as lung cancer is the major thoracic cancer which is the leading cause of cancer-related death in the world and only have an overall 5-year survival rate of about 20% (32), it leaves a huge gap for new treatments to fill thus reducing the health burden. With its high incidence and mortality, it is of great fundamental and clinical significance to further explore the regulatory mechanism inside the tumor microenvironment (TME) in order to find new biomarkers that can accurately predict the response to ICT.

It has been well established that TME has been significantly changed by altered tumor energy metabolism, leading to complexity for cancer treatments. The reprogramed glucose metabolism in cancer cells have been elegantly summarized in many reviews to explore the potential direct therapeutic opportunities targeting the metabolism pathways (33–35). Indirectly, the altered metabolism will change many metabolite concentration, and these metabolites have been identified to serve as signaling molecules; recent studies have shown that they are substrates for several newly identified modifications such as succinylation (36), hydroxybutyrylation (37), lactylation (38, 39), crotonylation (40), and many others (41). While these metabolic modifications are not in the scope of this review, the focus of our discussion is on glycosylation and palmitoylation, which use saccharide and saturated fatty acid as substrates for protein modifications. Glucose from extracellular sources is uptaken by cells and degraded to provide energy mainly by the anaerobic and aerobic pathways of glycolysis [also known as tricarboxylic acid cycle (TCA)]. A branch pathway called hexosamine biosynthetic pathway (HBP) uses fructose-6-P (F6P) from glycosylation to produce a nucleotide sugar UDP-*N*-acetylglucosamine (UDP-GlcNAc), which is the key substrate used for the glycolysis of proteins (34, 42). In cancer cells, this HBP pathway is usually upregulated with increased glycolysis, leading to a higher level of protein glycosylation and also increases heterogenity (43). Intracellular palmitic acid either comes from endogenous fatty acids (FA) synthesized by fatty acid synthase (FASN; only happening in normal liver and adipose tissue), or from exogenous fatty acids supplied by CD36-mediated uptake. It is well documented that cancer cells also have a high demands for lipids *via* increased FA synthesis (44). Palmitic acid (PA) not only provides energy to cells by β-oxidation but also serves as a substance involved in palmitoylation (45). However, protein palmitoylation level is not only determined by PA concentration but also by the specific palmitoyl *S*-acyltransferases ZDHHC enzyme which catalyzes the reaction, thus creating a complicated situation in cancer cells (46).

Like many other classic posttranslational modification (PTM) such as methylation, acetylation, phosphorylation, ubiquitination, etc., glycosylation and palmitoylation modulate protein characteristics such as structure, activity, subcellular localization, stability, and protein-protein interactions. Glycosylation involves in many fundamental molecular and cell biological processes such as cell-cell adhesion, cell-matrix interaction, cell metabolism and signaling, immune surveillance, tumor angiogenesis, etc. (43) Since palmitoylation adds a 16-carbon fatty acyl group onto cysteine side chain of protein, other than affecting the innate properties of target proteins which have critical roles in inflammation and cancer development, it most commonly increases membrane binding of target proteins and also manipulates the membrane properties. Palmitoylation plays an important role in many immunological processes such as inflammation signaling, phagocytosis, endothelial and epithelial integrity, etc (47). An increasing body of evidence showed that glycosylation and palmitoylation play important roles in tumor immunology especially PD-1/PD-L1 pathway and inflammation. In this review, we summarize the recent progresses on how these two metabolic modifications regulate immune checkpoint and inflammatory effect, aimed at discovering new cancer targets in TME for more accurate individual cancer treatments.

## The Metabolic Modifications in Tumor Microenvironment

The tumor microenvironment (TME) is a complicated ecology consisting of many cells that co-evolve with cancer cells to influence the development and progression of cancer and noncellular components. In addition to malignant cells, there are many cell types including nutritional supportive cells such as stromal cells, endothelial cells, adipocytes, fibroblasts, tumor vasculature, and immune cells such as lymphocytes, dendritic cells, cancer-associated fibroblasts, macrophage, platelets, and others. Malignant cells have accumulated enough mutations to acquire the tumor hallmarks including growth, immune suppression, immortality, tumor-promoting inflammation, invasion and metastasis, angiogenesis, genome instabilities, dysregulated metabolism, etc. (48). There are various complex cell-cell communications inside TME, thus many cells reprogrammed their metabolic pathways to adapt to the acidic, hypoxic environment to provide energetic support to cancer cells, to help cancer cells evade immune surveillance, to help malignant cells to spread and invade, and so on. The extracellular matrix (ECM) in TME which is composed of active tissue components such as glycoproteins, collagens, and enzymes can influence cell adhesion, proliferation, communication, and migration (49). The composition and structure of the TME vary among cancer types and among patients, contributing to tumor heterogeneity which affects the response to cancer treatments. In the following, we focus on the most recent progresses on how the metabolic changes of lipids and carbohydrates affect immunosuppression and inflammation in TME.

Cell metabolism has been well connected to cancer a century ago since Otto Warburg discovered the aerobic glycolysis in cancer, known as the Warburg effect (50). Compared with normal cells, cancer cells develop a great capacity to grow, proliferate, and survive under stress conditions, which need a lot of energy and materials for membrane formation, biomass accumulation, immune suppression, etc. They modify several pathways in the metabolism of carbohydrates, lipids, proteins, and nucleotides, thus each cell types have a specific set of enzymes and metabolites to achieve a favorable microenvironment. Over the past 20 years, there is growing evidence that metabolism is closely linked to the development of tumor and tumor microenvironment. For example, it has been well established that cancer cells use hyperactivated PI3K-AKT pathway (activated by hormone insulin signal) to drive glucose uptake by upregulating glucose transporters GLUT1; this favors glycolysis pathway and stimulates a large amount of pyruvate and lactate production (51, 52), leading to acetyl-coenzyme A formation which could be used for ATP synthesis or for *de novo* lipogenesis (53, 54). On the other hand, the increased glutamine and glucose uptake by cancer cells and immune cells (55, 56) not only contributes to increased glycolysis but also increases flux into the metabolic branch HBP pathways, and the end‐product of HBP is uridine diphosphate (UDP)‐GlcNAc, which is a critical metabolite used for *O*-GlcNAcylation as well as for *N*‐glycosylation (57). Actually, there is direct evidence demonstrating that (UDP)‐GlcNAc increased 12 times in breast cancer (58).

As cancer cells are rapidly proliferating cells, they need to form a large amount of new membrane, thus they have increased endothelial lipid *de novo* synthesis, which is referred as reprogramming of fatty acid metabolism (55). Actually, studies have shown that FASN (catalyzes the synthesis of palmitate) was elevated in many human cancers (59, 60). Acetyl-coenzyme A is also the substrate for the *de novo* fatty acid synthesis; FASN converts dietary carbohydrates to long-chain saturated fatty acids through acetyl-CoA (61), resulting in the accumulation of palmitate acid in tumor cells. In addition, cancer cells increase the expression of transmembrane proteins responsible for the uptake of exogenous FA, including CD36 (also known as FAT), the fatty acid transporter family (FATP), or the soluble carrier protein family 27 (SLC27) (62). Fatty acids are major components of cell membrane; saturated and monounsaturated fatty acids are biomarkers in several types of cancer, thus they open interesting perspectives for biomarker discovery and nutritional strategies to control cancer. Palmitic acid is the most abundant lipid acids in cells (20%~30% of total fatty acids) with a concentration ranging from 0.3 to 4.1 mM (63); it is strictly regulated, and abnormal concentration increase will trigger higher level of protein lipidation such as palmitoylation. The growing understanding in glycobiology and lipid metabolism has been summarized in many other review papers (43, 64–66). As many PTM are sensitive to the concentration of their metabolite substrate, they are tightly linked to and regulated by cell metabolism; this is beyond the scope of this review but well reviewed elsewhere (67). Next, we only discuss how glycosylation and palmitoylation affect inflammation and cancer immunotherapy.

## Glycosylation and Palmitoylation on Tumor Immune Checkpoint

Immunotherapies using checkpoint inhibitors tend to shrink tumors in patients with different cancers and are linked to durable responses and low toxicity levels. Therefore, they are regarded as promising interventions that can help in managing cancer. The PD-1/PD-L1 axis and cytotoxic T-lymphocyte-associated protein 4 (CTLA4), the two most well-characterized immune checkpoint signaling pathway to date, utilize quite different mechanisms to hinder T-cell–dependent immunity. Previous research has established that CTLA4 primarily prevents T-cell activation. Activation of T cells requires two signals, interaction between T-cell receptor (TCR) and the antigen-MHC complexes on the antigen-presenting cell (APC) surface and the engagement of costimulatory molecule CD28 with B7 molecules on APC (4). Upon activation, T cells proliferate and differentiate, and they express immune checkpoints such as CTLA-4 and PD-1. CTLA4 outcompetes CD28 for binding to B7 molecules and abrogate T-cell responses (68, 69). Different from CTLA4, the PD-1 association with PD-L1 plays an inhibitory function mediated by the tyrosine phosphatase SHP-2, which modulates signaling molecules downstream of the TCR binding with antigen-MHC I complexes, thus suppressing T-cell cytotoxic effect against target cells and leads to T-cell exhaustion and death (70, 71). It becomes evident that overexcessive immune response is repressed by these regulatory mechanism aiming at protecting organism from self-immune disease.

Tumor ICT, also known as tumor immune checkpoint blockade, becomes a new treatment for cancer since the first antibody targeting CTLA-4 was discovered by Nobel laureate James P. Allison (72, 73). Different from radiotherapy, chemotherapy, or targeted therapy that directly attack certain tumor cells, ICT leverages the cytotoxic potential of the immune system against cancer cells. Through blocking inhibitory regulation pathway by harnessing antibody, ICT unleash T-cell antitumor response. Unfortunately, the protective inhibitory mechanism is hijacked by various types of tumor cells to attenuate T-cell response and escape antitumor immune surveillance. Further research on the mechanism of *de novo* and acquired resistance to ICT and effective judgment on which cohort of patients are suitable for the use of ICT will lay a foundation for combined pharmacotherapy to treat cancer. In multiple mouse models, pharmacological inhibition of oncogenic signaling pathways, such as Wnt signaling pathway, CDK4-CDK6-dependent cell cycle, and MAPK signaling pathway can result in reversal resistance to anti-PD1 therapy. Moreover, combination therapy with anti-CTLA4 plus anti-PD1 monoclonal antibodies leads to the enhancement both effector CD4+ and CD8+ T-cell responses (72). Taken together, fundamental research in this field not only holds promise in broadening the impact and efficacy of immune checkpoint blockade but also attenuates immune-related adverse events (74). Among many ways to tackle the problems in the field, we focus on how two metabolic modifications, glycosylation and palmitoylation, impact protein trafficking, folding, and stability, ultimately altering its biochemical and biophysical properties to influence immunosuppression and inflammation. The modifications we discussed are summarized in **Table 1**.

**Table 1:** Glycosylation and palmitoylation associated with adaptive immunity.

Modification types	Protein	Catalyzing enzyme	Modification sites	Cell types	Mechanism	References
Glycosylation	PD-L1	B3GNT3	N192, N200, and N219	TNBC	EGFR signaling downstream TCF4 upregulates B3GNT3 to catalyze glycosylation in TNBC, attenuating the association with GSK3β and preventing 26S proteasome degradation of PD-L1 by β-TrCP	(75)
	PD-L1	STT3A	N35, N192, N200, and N219	TNBC	Epithelial–mesenchymal transition upregulates STT3A for the subsequent PD-L1 glycosylation in TNBC, preventing PD-L1 degradation by lysosome	(76)
	PD-L1	GLT1D1		B-Cell lymphoma	GLT1D1 enhances *N*-glycosylation and stability of PD-L1 protein	(77)
	PD-1	FUT8		Lung adenocarcinoma	Unknown	(78)
	PD-1		N58		Enhancing the interaction between PD-1 an PD-L1	(79)
Palmitoylation	PD-L1	DHHC3	C272	Breast cancer	Regulating PD-L1 stability	(80)
	PD-L1	DHHC9	C272	Colorectal cancer	DHHC9-mediated PD-L1 palmitoylation suppresses the mono-ubiquitination of PD-L1, thereby preventing its trafficking to the MVB by ESCRT followed by blocking the lysosomal degradation of PD-L1	(81)

## Glycosylation on PD-L1

Protein glycosylation is characterized as a universal PTM that shows some glycans are covalently attached to a polypeptide backbone *via* glycosidic bond by glycosyl transferase. According to which residue is added, glycosylation can be divided into *O*-glycosylation and *N*-glycosylation (82). *O*-glycosylation takes place in the Golgi apparatus. It is mostly initiated by linking a single *N*-acetylgalactosamine (GalNAc) to Ser or Thr residue, and usually can be extended by fucosylation and sialylation resulting in producing various “cores” and different terminal structures (83). In contrast, *N*-glycosylation occurs on *N*-acetylglucosamine (GlcNAc) linked to the consensus NXT motif (Asn-X-Ser/Thr); it is initiated in the endoplasmic reticulum and finished in the Golgi apparatus. It shares a common pentasaccharide core region that can be further modified by GlcNAc, Gal, and sialic acid as terminal structures (84). Glycans dictate proteolysis patterns and directly mediate ligand-receptor interactions, oncogenic signaling transduction, immune recognition, migration, and both cell-cell and cell-matrix adhesions.

**Figure 1:**
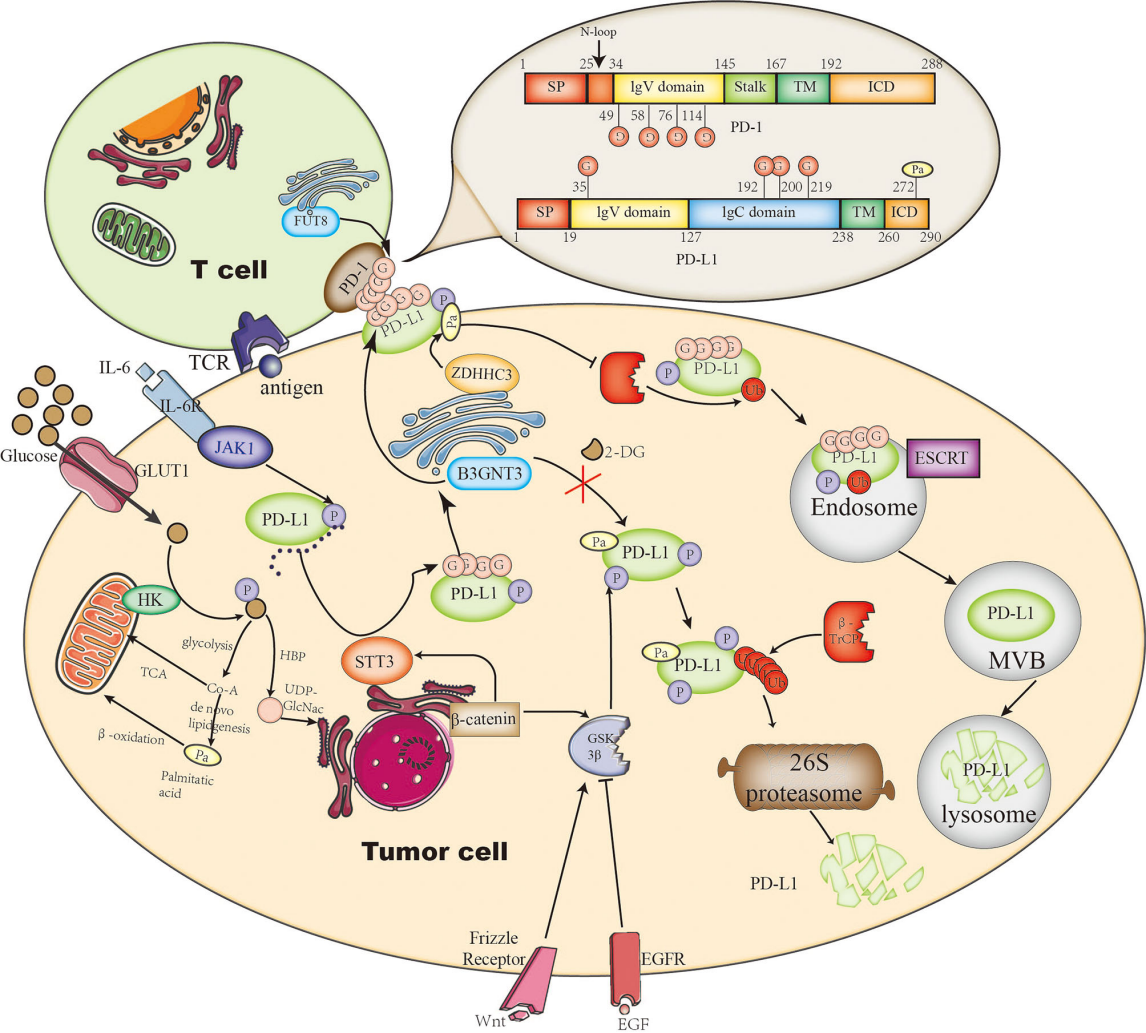
The roles of glycosylation and palmitoylation of PD-1/PD-L1. In T cells, PD-1 is glycosylated by FUT8, leading to its stable expression on the cell surface and causing a stronger interaction with glycosylated PD-L1. On the other hand, PD-L1 is glycosylated or degraded through different pathways. It can be glycosylated by STT3 in ER or by B3GNT3 in the Golgi apparatus. Glycosylation helps to maintain its stability by preventing GSK3β-dependent phosphorylation and downstream β-Trcp-mediated 26S proteasome degradation. In addition, PD-L1 palmitoylation catalyzed by ZDHHC3 stabilizes PD-L1 through blocking PD-L1 mono-ubiquitination, otherwise ubiquitinated PD-L1 will be sorted by endosomal sorting complexes required for transport (ESCRT) to the multivesicular body (MVB) for degradation. Glycosylation is affected by the branch glucose metabolism pathway HBP, which directly provides the glycosylation substrate (UDP)-GlcNAc. Palmitoylation is affected by the lipid synthesis which leads to palmitate acid storage.

PD-L1 is a single-pass type-I membrane protein generally expressed on the cellular membrane in tumor cells and also on the host immune cells. By interacting with its receptor PD1 through its extracellular domain, PD-L1 blocks effective T-cell attacks. It has been identified by mass spectrometry and sequence analysis that PD-L1 is principally modified by *N*-glycan at four glycosylation sites (N35, N192, N200, N219) in the extracellular domain (85); these modifications are predominantly correlated with its stability and interaction with PD1, representing the functional form of PD-L1. At the earliest in 2016, the Hung group discovered that PD-L1 *N*-glycosylation enhances its stability by abolishing the interaction with GSK3β, which can phosphorylate PD-L1 at T180 and S184 sites thus mediating 26S proteasome degradation of PD-L1 by β-TrCP (85). Consistently, mutations of PD-L1 at three *N*-glycosylation sites (N192, N200, N219) attenuate its association with GSK3β and decrease its protein level. They further identified that PD-L1 *N*-glycosylation is catalyzed by β-1,3-*N*-acetylglucosaminyltransferase3 (B3GNT3), which is initiated by EGFR signaling downstream the transcription factor TCF4, and boosts the interaction with PD-1 in triple-negative breast cancer (TNBC) (75). Accordingly, knockout of B3GNT3 in TNBC diminishes PD-L1 *N*-glycosylation and prevents tumor growth. The glycosylation stabilizes PD-L1 expression not only *via* EGFR signaling but also by epithelial–mesenchymal transition (EMT) in TNBC. EMT transcriptionally upregulates *N*-glycosyltransferase STT3 by Wnt/β-catenin pathway, thus leading to elevated PD-L1 *N*-glycosylation in cancer stem-like cells (86). In this pathway, PD-L1 is phosphorylated at Y112 by IL-6-activated JAK1, then recruits STT3A for the subsequent PD-L1 glycosylation (76) (**Figure 1**). However, the correlation between B3GNT3-dependent glycosylation and STT3-mediated glycosylation remains an open question.

PD-L1 has been found glycosylated in many cancer types including breast cancer, melanoma, lung cancer, colon cancer, etc. (85), suggesting a universal feature for all cancers. However, the glycosylation pathway could be cancer-type dependent (through particular glycosyltransferases). For example, in some subtypes of B-cell lymphoma, glycosyltransferase 1 domain-containing 1 (GLT1D1) is significantly upregulated and enhances PD-L1 stability through *N*-glycosylation; downregulation of GLT1D1 caused a decrease in glycosylated PD-L1 protein, leading to an increase in cytotoxic T-cell infiltration in the tumor microenvironment (77). It suggests that GLT1D1 probably is a promising biomarker for lymphoma; further studies on cancer-type–dependent glycosylation pathways are needed. Interestingly, drug-conjugated STM108, an antibody that is specially developed, recognizes glycosylated PD-L1 at N192 site and leads to PD-L1 internalization and degradation, inducing a potent antitumor effect as well as a bystander-killing effect on adjacent cancer cells lacking PD-L1 expression without any detectable toxicity (75). This suggests that targeting glycosylated PD-L1 is a potential strategy of immunotherapy, and the glycosylation pathways could also serve as targets or biomarkers for early diagnosis.

One of the biggest problems of ICT is how to accurately classify patients suitable for immunotherapy to achieve higher response rate. As PD-L1 is heavily glycosylated and glycosylation is heterogenous, it complicates the situation, because glycosylation not only stimulates interaction with PD-1 but also prevents binding to PD-L1 antibody (75). This has been proposed to illustrate why PD-L1–negative patients still have a favorable clinical response to ICT (87). By removing *N*-glycans from PD-L1, the Hung group discovered that deglycosylation significantly improves anti-PD-L1 antibody binding affinity, thus resulting in a more accurate PD-L1 quantification and prediction of clinical outcome in breast cancer (88). Deglycosylation can be achieved by using a glucose analog 2-deoxyglucose (2-DG) to block PD-L1 glycosylation and trap them inside the endoplasmic reticulum. 2-DG has been used to reverse PD-L1 expression on the cell surface of TNBC mediated by PARP inhibitor (upregulates PD-L1), and it has showed a potent antitumor activity (89). In addition, it enhances 4-1BB–mediated antitumor immunity *via* PD-L1 deglycosylation (4‐1BB engages with 4‐1BB ligand (4‐1BBL) or agonist antibody to stimulate CD8+ T-cell activity or NK cell growth) (90). Furthermore, it has been shown that the combination of 2-DG and EGFR tyrosine kinase inhibitor (TKI) gefitinib inhibits glycosylation of PD-L1 in TNBC. These studies suggest that 2-DG blocking PD-L1 glycosylation is a universal pathway and combination therapy could also be useful for lung cancer. Very interestingly, a more recent study confirmed this idea in lung cancer specimens (91). Next, we may use deglycosylation to provide a more reliable way to quantify PD-L1 level and guide anti-PD-1/PD-L1 immunotherapy.

## Glycosylation on PD-1

PD-1 is also a single-pass type-I membrane protein, unlike PD-L1, it is usually expressed on the cellular membrane of T lymphocyte. Upon T-cell activation, TCR-dependent signaling initiates the transcription of PD-1, and IFN-γ–mediated signaling causes durable PD-1 expression (92). Completely opposite to PD-L1, GSK3β facilitates PD-1 expression by inhibiting T-bet induction, which suppresses transcription of PD-1 along with B-lymphocyte–induced maturation protein 1 (Blimp-1) after initial transcription. Similar to PD-L1, four *N*-glycosylation sites on the extracellular domain of PD-1 (N49, N58, N74, and N116) have also been identified by mass spectrometry analysis; they are also associated with stable cell-surface expression of PD-1 (78). Okada et al. has identified Fut8 as the core fucosyltransferase to fucosylate PD-1 and positively regulates cell-surface PD-1 expression, thus inhibition of Fut8 reduces PD-1 cell-surface expression and promotes T-cell antitumor activity in melanoma (78). Similarly, Zhang et al. discovered that core fucosylation was significantly upregulated in lung adenocarcinoma and demonstrated that de-core fucosylation of PD-1 *via* Fut8 knockout enhances CD8^+^ cytotoxic T-lymphocyte (CTL) activation and cytotoxicity in lung adenocarcinoma (93). Furthermore, the glycosylation of PD-1 at N58 is critical for PD-1 cell-surface expression and stability and is essential to mediate its interaction with PD-L1 (79). Based on the glycosylation research on PD-1, an adenine base editor (ABE) induced mutation at PD-1 N74 site, downregulating PD-1 expression in CAR-T cells and enhancing CAR-T cell cytotoxic functions *in vitro* and *in vivo* (94) (**Figure 1**).

Very recently, a new monoclonal antibody STM418 targeting N58-glycosylated PD-1 was developed; it shows higher binding affinity to PD-1 than previous FDA-approved antibodies and induces much stronger T-cell antitumor immunity (79). This is not a unique instance, a co-crystal structure of PD-1 glycosylated at N58 with monoclonal antibody MW11-h317 Fab provides the structure basis for molecular interaction for PD-1 *N*-glycosylation recognization (95). Another newly developed PD-1 antibody, known as camrelizumab and currently undergoing phase II/III trials, also selectively binds to N58-glycosylated PD-1 to inhibit PD-1/PD-L1 pathway (96). These studies suggest that targeting glycosylated PD-1 may improve immunotherapy response.

Although we have known the *N*-glycosylation enzymes and sites on PD-1/PD-L1, the exact composition of glycome on PD-1/PD-L1 is elusive thus far; further details revealing glycome may disclose more potential molecular targets. Though biochemical experiments and animal models have demonstrated that target PD-1/PD-L1 *N*-glycosylation not only improves the accuracy of disease diagnosis but also the effectiveness of treatment, more clinical trials are needed to verify its efficacy and safety. Additionally, unlike *N*-glycosylation, less of the *O*-glycosylation enzymes and sites have been discovered on PD-1/PD-L1. GCNT3 is involved in *O*-glycosylation and impacts the clinical outcome of colon and ovarian cancers (97); however, it is unclear whether it could also affect PD-1/PD-L1 *O*-glycosylation in modulating immune checkpoint. The rapid progress in this field in both basic and clinical research will not only advance our ability to identify patients who are most likely to benefit from certain immunotherapy but also help to predict response in the clinic and even lead to new treatments.

## Palmitoylation on PD-L1

Palmitoylation is a lipidation process that covalently attaches a palmitate acid to residues on a protein *via* three different ways. (1) *S*-palmitoylation, linking to cysteine residues by thioester linkages, (2) *O*-palmitoylation, linking to serine/threonine residues by oxyester linkages, and (3) *N*-palmitoylation, linking to primary amino groups by amide linkages. The *S*-palmitoylation is the only reversible reaction which is catalyzed by endomembrane-bound palmitoyl acyltransferases (PATs); mammals comprise 23 distinct enzymes which contain the zinc finger DHHC-type domain, while very few protein palmitoylation is self-catalyzed (98–101). Contrary to a variety of PATs, only six depalmitoylation enzymes, APT1, APT2, PPT1, ABHD17A, ABHD17B, and ABHD17C, are reported to catalyze protein *S*-deacylation thus far (102). Emerging evidence has illustrated that *S*-palmitoylation regulates numerous biological processes through affecting protein migration, membrane localization, stability, and interactions (99, 103).

In 2019, two groups reported simultaneously that *S*-palmitoylation maintains PD-L1 stability and inhibits T-cell cytotoxic response (80, 81). Both of them identified C272 as the palmitoylation site on PD-L1, substituting C272 with alanine abolishes PD-L1 palmitoylation in tumor cells. Interestingly, the Hung group use breast tumor cells and find DHHC9 as the palmitoyl acyltransferases (80), while the Xu group identified DHHC3 as the PAT for PD-L1 in colorectal cancer (CRC) (81). The latter further proposed that the *S*-palmitoylation regulates PD-L1 stability by suppressing the mono-ubiquitination of PD-L1, thereby blocking the lysosomal degradation of PD-L1 *via* preventing its trafficking to the MVB by ESCRT, causing increased cell surface expression of PD-L1 and thus suppressing T-cell cytotoxicity (**Figure 1**). Interestingly, based on the idea that DHHC recognize their substrate by amino acid sequence, a competitive polypeptide is designed towards more inhibition specificity for PD-L1 than the commonly used universal palmitoylation inhibitor 2-BP, indeed the expression of PD-L1 was reduced by this inhibitor in tumor cells (81). This idea could be useful in developing research tools or clinical drugs for other palmitoylation targets; further research on improving specificity, stability, and efficacy of this kind of peptide inhibitors to target the palmitoylation in other malignant cancers such as lung cancer in encouraging.

## Innate Immunity, Inflammation, and Immunotherapy

The innate immunity consists of many types of cells and soluble molecules in tissues and blood that constantly prevent microbes from invading and eliminating other offending agents. The cellular sensors for pathogen- and damage-associated molecular patterns in innate immune cells are called pattern recognition receptors (PRRs). Five main classes of PRRs have been described: membrane-bound TLRs and CLRs, cytoplasmic NLRs and RLRs, and several DNA sensors such as cGAS (104, 105). Once activated, PRRs initiate multiple innate immune signaling pathways, leading to the production of type I interferons (IFN-I) and proinflammatory cytokines, which is referred to as inflammation (106). Considering that the human body does not develop a new set of PRRs for identifying cancer cells, it is usually accepted that antitumor immunity shares the same PRRs with the innate immunity machine.

Indeed, inflammation is a sophisticated process closely associated with a variety of tumors, which tends to form an inflammatory environment surrounding it. There is considerable evidence indicating that chronic inflammation plays a critical role for tumorigenesis, for example, colorectal cancer can develop on the ground of inflammatory bowel disease (IBD) (107), chronic hepatitis B greatly increases the risk of hepatic carcinoma (108), and even lung cancer caused by smoking is attributed to irritant-induced chronic inflammation, not genetic mutations (109). The inflammatory cells recruited to the tumor microenvironment releases a majority of cytokines, which support the survival and proliferation of tumor cells (110–112). Nonetheless, it is controversial how inflammation influences the outcome of ICT, because some articles reported inflammation promotes the activation of T cell but others were opposite (113–115). Next, we will discuss the molecular mechanisms of how palmitoylation regulates inflammation.

## Palmitoylation Regulates Innate Immunity and Inflammation

**Figure 2:**
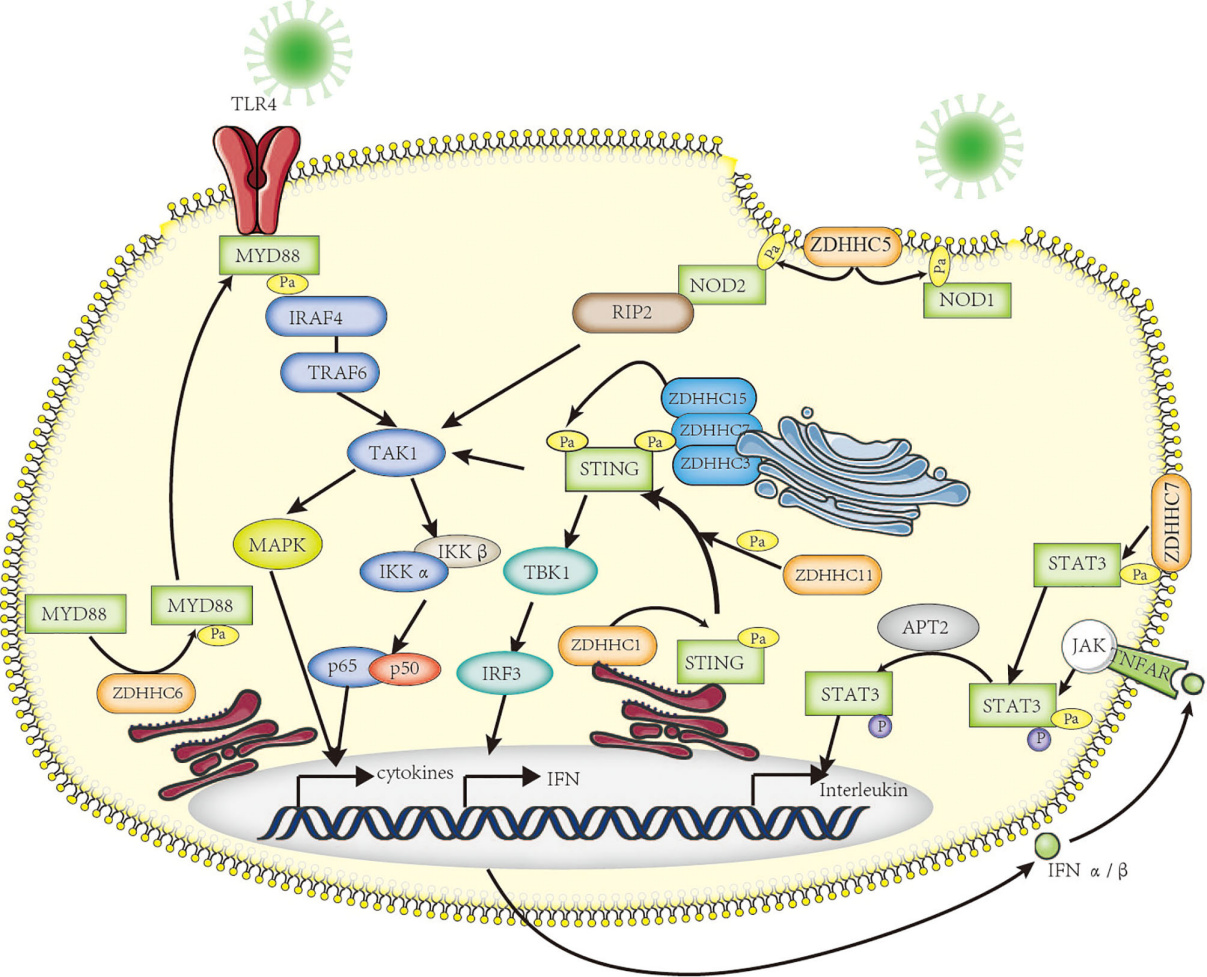
The roles of palmitoylation in inflammation. Palmitoylation affects inflammatory response in many ways. MYD88 is palmitoylated by ZDHHC6, facilitating downstream NF-κB inflammation pathway activation by mediating association with IRAF-4. NOD1/2 is palmitoylated by ZDHHC5, which is required for membrane recruitment and downstream signaling. Five palmitoyl-transferases (localized at different cell compartments including ER and Golgi apparatus) positively regulate STING-dependent inflammatory response. The STAT3 palmitoylation–depalmitoylation cycle, catalyzed by DHHC7 and APT2, controls phosphorylation of STAT3 by JAK2 and contributes to STAT3 nuclear translocation, which is essential for IL-17–induced inflammation.

TLR4, a class of pattern recognition receptor in sentinel cells of innate immunity such as macrophage, dendritic cells, and neutrophils, belongs to the Toll-like receptor family. TLR4 recognizes LPS of gram-negative bacteria and is involved in inflammasome activation through activating downstream NF-kB signaling to release inflammatory factors TNF-α, IL-1β, and IL-18 (116). Earlier in 2016, Wei et al. have shown that FASN is indispensable for diet-induced inflammation, suggesting fatty acid metabolism plays a possible role in modulating inflammatory response (117). A series of follow-up research has proven that the saturated fatty acid palmitate is not a TLR4 agonist but participates in palmitoylation of C113 on MYD88, a downstream of TLR4, and this palmitoylation promotes MYD88 association with IRAF4 to activate the downstream NF-κB signaling pathway, thus inducing altered cellular metabolism and inflammation in neutrophils (118, 119) (**Figure 2**). Pharmacological inhibition of MYD88 palmitoylation in neutrophils suppressed TLR-induced inflammation and enhanced the chemotactic activity of neutrophils, improving the survival of mice with sepsis.

NOD1 and NOD2, another class of intracellular pattern-recognition receptors in macrophage, belong to the NOD-like receptor family that recognizes peptidoglycans associated with microorganisms and is involved in host defense through activating downstream NF-kB and p38 MAPK signaling to release chemokine CXCL-1 and inflammatory factor IL-6 (120). It has been known that dysregulation of NOD1/2 function leads to severe immunologic and inflammatory diseases such as Crohn’s disease (CD) and Blau syndrome (121). The Neculai group provided substantial evidence showing that NOD1/2 are *S*-palmitoylated by ZDHHC5, and this *S*-palmitoylation is required for membrane localization and to induce NF-kB signaling in response to peptidoglycans (122) (**Figure 2**).

STING is a central adaptor in innate immune responses to DNA viruses, which are recognized by cytosolic DNA sensor cGAS leading to secretion of type I IFNs through the activation of TANK-binding kinase 1 (TBK1) and, subsequently, of the transcription factor IFN regulatory factor 3 (IRF3) and nuclear factor kappa B (NF-κB) (123, 124). It has been reported that STING can be regulated by ER-associated ZDHHC1 (125) and ZDHHC11 in innate immune responses against DNA viruses (126). However, Mukai et al. demonstrated that STING is palmitoylated in the Golgi apparatus at C89/91 by ZDHHC3, ZDHHC7, and ZDHHC15; this modification is essential for STING activation and the subsequent activation of IFN-β or NF-κb (127). More interestingly, covalently bound small-molecule inhibitors targeting STING palmitoylation was developed recently; they further confirmed that palmitoylation of STING is essential for its assembly into multimeric complexes at the Golgi apparatus and the activation of downstream STING-triggered inflammatory signaling (128) (**Figure 2**).

One more advanced progress on palmitoylation studies is the STAT3 palmitoylation–depalmitoylation cycle. The palmitoylation on Cys108 of STAT3 catalyzed by DHHC7 promotes membrane recruitment and phosphorylation by JAK2, then Acyl protein thioesterase 2 (APT2) depalmitoylates phosphorylated STAT3, this leads to nuclear translocation of p-STAT3, which eventually facilitates STAT3-mediated IL-17 transcription and differentiation of TH17 cells (129). Dysfunctional differentiation of TH17 cells has an important pathogenic role in IBD, including ulcerative colitis and Crohn’s disease; therefore, both DHHC7 and APT2 could be new therapeutic targets for IBD treatment.

All these palmitoylation and depalmitoylation examples (**Table 2**) underscore the direct evidence that palmitoylation regulates innate immunity and inflammation. Given that TME has altered metabolism pathways for lipid synthesis and causes a higher palmitate concentration, it is reasonable to speculate that at least in some circumstance a higher level of palmitoylation on cancer-related protein targets occurs, thus positively regulating tumor survival and immune suppression. Tumor cells secrete many immunosuppressive factors to directly alter T-cell effector functions and inhibit innate immune cells, preventing them from sustaining efficient antitumor immune responses. Additionally, inflammatory immune cells in TME such as tumor-associated macrophage generate a tolerant environment that suppresses T-cell response, thereby leading to immune escape. Understanding the palmitoylation-mediated regulatory mechanism of inflammation activation and tumor evasion will lead to further understand how cancer immunotherapy can be manipulated. Thus, targeting palmitoylation to regulate inflammation could provide new ways for cancer treatments.

**Table 2:** Palmitoylation and depalmitoylation related to inflammation.

Modification types	Protein	Catalyzing enzyme	Modification sites	Cell types	Mechanism	References
Palmitoylation	MyD88	DHHC6	C131	Neutrophils	DHHC6-mediated MyD88 palmitoylation promotes the MYD88 association with IRAF4 and downstream NF-κB signaling pathway activation	(119)
	NOD1	DHHC5	C558, C567, and C952	BMDMs	DHHC5-catalyzed NOD1 palmitoylation is required for their membrane localization and ability to induce NF-kB signaling in response to C12-iE-DAP	(122)
	NOD2	DHHC5	C395 and C1033	BMDMs	DHHC5-catalyzed NOD2 palmitoylation is required for their membrane localization and ability to induce NF-kB signaling in response to MDP	(122)
	STING	DHHC1		BMDMs	ZDHHC1 is a positive regulator *via* mediating aggregation of STING and recruitment of the downstream signaling components TBK1 and IRF3	(125)
	STING	DHHC11		BMDMs	ZDHHC11 is a positive regulator facilitating the optimal recruitment of IRF3 to STING	(126)
	STING	DHHC3, DHHC7, and DHHC15	C89/C91	BMDMs	Palmitoylation of STING is essential for its assembly into multimeric complexes at the Golgi apparatus and the activation of downstream STING-triggered inflammatory signaling	(128)
	STAT3	DHHC7	C108	Th17	STAT3 palmitoylation promotes membrane recruitment and phosphorylation by JAK2	(129)
Depalmitoylation	STAT3	APT2	C108	Th17	APT2 depalmitoylates phosphorylated STAT3, facilitating nuclear translocation of p-STAT3	(129)

## New Therapeutic Opportunities

ICT revolutionized the oncology field and opened a new door with the hope of curing cancer. It has achieved great success in some cancer types such as melanoma; however, in some other cancer types such as lung cancers, ICT is still facing great challenges because of their unpredictable responses and the risk of relapse. The regulatory mechanisms of PTMs in tumor cells and tumor microenvironment have been a hot research topic for several decades and achieved great progress in the last decade. The basic research in this field disclosed more secrets of tumor heterogeneity in TME, revealing many regulatory mechanisms on tumorigenesis and immune suppression and suggesting a number of new ways for cancer treatments, especially for those cancers with a low response rate or low survival rate, such as lung cancer. It is promising to target many components in glycosylation/deglycosylation and palmitoylation/depalmitoylation pathways; this includes the upstream material acquiring or biosynthesis pathways, the direct glycotransferases, palmitoylases/depalmitoylases, and many downstream effector proteins such as PD-1/PD-L1, NOD1/2, STAT3, etc. Due to the urgent need to achieve a more predictable immunotherapy outcome and benefit from more advanced research methods on glycosylation and palmitoylation, we expect more groundbreaking findings in this area, which will improve the outcomes of current immunotherapies *via* stratifying cancer patients or combinational therapies and provide more new therapeutic opportunities. In this regard, in the era of “big data,” a more accurate precision cancer immunotherapy is just around the corner.

## Author Contributions

SZ and PZ wrote the review. QS discussed the review. All authors proofread the review. All authors contributed to the article and approved the submitted version.

## Conflict of Interest

The authors declare that the research was conducted in the absence of any commercial or financial relationships that could be construed as a potential conflict of interest.

## Publisher’s Note

All claims expressed in this article are solely those of the authors and do not necessarily represent those of their affiliated organizations, or those of the publisher, the editors and the reviewers. Any product that may be evaluated in this article, or claim that may be made by its manufacturer, is not guaranteed or endorsed by the publisher.
